# DKK1 loss promotes endometrial fibrosis via autophagy and exosome-mediated macrophage-to-myofibroblast transition

**DOI:** 10.1186/s12967-024-05402-5

**Published:** 2024-07-03

**Authors:** Zhanqin Zhang, Jianguo Hu

**Affiliations:** 1 Department of Anesthesiology, The First Affiliated Hospital of Xi’an Jiaotong University, Xi’an, China; 2 Department of Obstetrics and Gynecology, Second Affiliated Hospital, Chongqing Medical University, Linjiang Road, No. 76, Chongqing, 400010 China

**Keywords:** DKK1, Autophagy, IL-8, Intrauterine adhesions, Wnt, β-catenin

## Abstract

**Introduction:**

Intrauterine adhesions (IUA) manifest as endometrial fibrosis, often causing infertility or recurrent miscarriage; however, their pathogenesis remains unclear.

**Objectives:**

This study assessed the role of Dickkopf WNT signaling pathway inhibitor 1 (DKK1) and autophagy in endometrial fibrosis, using clinical samples as well as in vitro and in vivo experiments.

**Methods:**

Immunohistochemistry, immunofluorescence and western blot were used to determine the localization and expression of DKK1 in endometrium; DKK1 silencing and DKK1 overexpression were used to detect the biological effects of DKK1 silencing or expression in endometrial cells; DKK1 gene knockout mice were used to observe the phenotypes caused by DKK1 gene knockout.

**Results:**

In patients with IUA, DKK1 and autophagy markers were down-regulated; also, α-SMA and macrophage localization were increased in the endometrium. DKK1 conditional knockout (CKO) mice showed a fibrotic phenotype with decreased autophagy and increased localization of α-SMA and macrophages in the endometrium. In vitro studies showed that DKK1 knockout (KO) suppressed the autophagic flux of endometrial stromal cells. In contrast, ectopic expression of DKK1 showed the opposite phenotype. Mechanistically, we discovered that DKK1 regulates autophagic flux through Wnt/β-catenin and PI3K/AKT/mTOR pathways. Further studies showed that DKK1 KO promoted the secretion of interleukin (IL)-8 in exosomes, thereby promoting macrophage proliferation and metastasis. Also, in DKK1 CKO mice, treatment with autophagy activator rapamycin partially restored the endometrial fibrosis phenotype.

**Conclusion:**

Our findings indicated that DKK1 was a potential diagnostic marker or therapeutic target for IUA.

**Supplementary Information:**

The online version contains supplementary material available at 10.1186/s12967-024-05402-5.

## Introduction

Intrauterine adhesions (IUA), also known as Asherman syndrome, often lead to infertility and recurrent miscarriage. The current treatment effect of intrauterine adhesions is not very satisfactory [1]. About 1 in 5 women experiencing a miscarriage suffers from this disorder [2]. Endometrial fibrosis is the histopathological manifestation of IUA, and abortion or infection is the most common cause of IUA [3, 4]. The current pathogenesis of IUA remains unclear, making the clinical treatment of IUA quite challenging.

Autophagy primarily involves the degradation of dysfunctional cellular components by lysosomes. In physiological state, autophagy can degrade damaged organelles, proteins and other biological macromolecules. These degradation products can provide raw materials for cell renewal. However, excessive autophagy is associated with diseases such as cancer, inflammation and fibrosis [5, 6]. There is a specific relationship between autophagy and fibrotic diseases. E.g., autophagy is a crucial regulator in pulmonary, cardiac, liver, and kidney fibrosis [7–10]. Autophagy deficiency induced by Sonic Hedgehog or DIO2 signaling can cause IUA, and autophagy activators can delay the occurrence of IUA [11, 12]; however, the mechanism of autophagy in intrauterine adhesion diseases still needs to be further studied.

In the present research, we employed next-generation sequencing technology to analyze the transcriptomic transcriptome of the endometrium of patients with IUA. Through the sequencing data of uterine adhesion tissue and normal endometrial tissue, we found that DKK1 was significantly down-regulated in uterine adhesion. As a classic inhibitor of WNT/ β-catenin signaling pathway, DKK1 plays an important role in fibrotic diseases. Therefore, this study chooses DKK1 as a further study. Our results indicated that DKK1 expression was decreased in endometrium of intrauterine adhesion compared with normal endometrium. Moreover, DKK1 regulated autophagy through WNT/β-catenin and PI3K/AKT/mTOR signaling cascades. Decreased DKK1 promoted the secretion of exosomes from stromal cells, thereby promoting macrophage-to-myofibroblast transition (MMT) and leading to IUA. Our study identifies a new therapeutic target of IUA.

## Materials and methods

### Study design

The transcriptomic differences between the endometrium of normal and IUA patients were analyzed by bulk RNA sequencing. Downregulation of DKK1 and autophagy markers were found in the endometrium of patients with intrauterine adhesion, along with up-regulation of fibrosis marker α-SMA and recruitment of macrophages at the adhesion site. This study subsequently established evidence in cell lines and transgenic mice to support the hypothesis that DKK1 is a promising target of treatment for IUA. The function of DKK1, rapamycin, and interleukin-8 inhibitors in modifying the phenotype of endometrial fibrosis has also been identified.

All in vitro experiments were repeated thrice. C57BL/6J and Dkk1fl/fl, Cre+/- mice were derived from the Cyagen Model Animal Research Center for mouse experiments. All procedures were approved by the animal ethics committee of the Second Affiliated Hospital of Chongqing Medical University(2023 − 700). One researcher was responsible for randomizing the animals, petri dishes, and slides; the other performed the blind data analysis. Each cage with 4–5 mice was housed in a pathogen-free mouse facility with free access to food and water on a 12-hour light/dark cycle. Based on the design of previous studies, the sample size for all experiments was 5 animals per group. The feeding environment of all mice included: relative humidity of 50 ± 1%, temperature of 22 ± 1 ℃, light / dark cycle of 12 h. All mice were studied in accordance with the regulations and guidelines of institutional animal care of Chongqing Medical University and the guidelines of AAALAC and IACUC.

### Endometrial tissues of normal and IUA patients

This study was approved by the Ethics Committee of the Second Affiliated Hospital of Chongqing Medical University, and all participants signed the written informed consent. Endometrium was collected from patients with late proliferative tubal infertility (normal control) and childbearing women diagnosed with intrauterine adhesions through hysteroscopy between April 2020 and October 2020. A total of 39 patients were enrolled, including 19 normal controls and 20 IUA patients (Table 1). The endometrial biopsy specimens were stored in a liquid nitrogen tank, followed by qPCR, western blot and immunohistochemistry detection. Three normal endometrial specimens and three intrauterine adhesions specimens were performed by RNA-seq detection. None of the participants had taken estrogen or progesterone for two months before sampling.

**Table 1:** Demographic information of the normal and IUA patients

Case	Age	Diagnosis	Childbearing history
Case 1	23	Severe intrauterine adhesions	G1P0
Case 2	25	Severe intrauterine adhesions	G2P0
Case 3	28	Severe intrauterine adhesions	G2P1
Case 4	28	Severe intrauterine adhesions	G3P0
Case 5	29	Severe intrauterine adhesions	G3P1
Case 6	25	Severe intrauterine adhesions	G1P0
Case 7	24	Severe intrauterine adhesions	G1P0
Case 8	25	Severe intrauterine adhesions	G1P0
Case 9	24	Severe intrauterine adhesions	G1P0
Case 10	31	Severe intrauterine adhesions	G2P0
Case 11	32	Moderate intrauterine adhesions	G2P1
Case 12	35	Moderate intrauterine adhesions	G1P0
Case 13	27	Moderate intrauterine adhesions	G4P1
Case 14	26	Moderate intrauterine adhesions	G4P0
Case 15	27	Moderate intrauterine adhesions	G1P0
Case 16	23	Mild intrauterine adhesions	G1P0
Case 17	25	Mild intrauterine adhesions	G2P1
Case 18	24	Mild intrauterine adhesions	G1P0
Case 19	34	Mild intrauterine adhesions	G1P0
Case 20	24	Mild intrauterine adhesions	G2P1
Case 21	23	Fallopian tube infertility	G1P0
Case 22	25	Fallopian tube infertility	G0P0
Case 23	25	Fallopian tube infertility	G0P0
Case 24	26	Fallopian tube infertility	G0P0
Case 25	28	Fallopian tube infertility	G0P0
Case 26	27	Fallopian tube infertility	G2P1
Case 27	29	Fallopian tube infertility	G1P1
Case 28	32	Fallopian tube infertility	G2P0
Case 29	34	Fallopian tube infertility	G3P1
Case 30	28	Fallopian tube infertility	G0P0
Case 31	29	Fallopian tube infertility	G2P0
Case 32	32	Fallopian tube infertility	G1P0
Case 33	27	Fallopian tube infertility	G0P0
Case 34	28	Fallopian tube infertility	G1P0
Case 35	31	Fallopian tube infertility	G2P1
Case 36	26	Fallopian tube infertility	G1P0
Case 37	33	Fallopian tube infertility	G3P2
Case 38	29	Fallopian tube infertility	G3P1
Case 39	28	Fallopian tube infertility	G2P1

### Next-generation sequencing technology

The total RNA of endometria of patients or mice was extracted by RNeasy Plus Micro Kit (Qiagen,74,134) according to the manufacturer’s instructions. RNA quality was checked using 1% agarose gel electrophoresis and RNA Nano 6000 Assay Kit (Agilent, 5067–1511). Shanghai Lifegenes Technology Co., Ltd. participated in library construction, sequencing and data analysis. GO enrichment analysis of differentially expressed genes was implemented using the clusterProfiler R package (v3.12.0). GO terms with P-value less than 0.05 were considered significantly enriched by differential expressed genes.

We used KOBAS v3.0 software to test the statistical enrichment of differential expression genes in KEGG pathways. KEGG terms with P-value less than 0.05 were considered significantly enriched by differential expressed genes.

### Protein extraction and Western blotting

The steps of Western blotting refer to our previous research. Briefly, the steps include: protein extraction, SDS-PAGE electrophoresis, membrane transfer, immunoreaction blocking, immunoreaction and chemiluminescence. The protein bands were measured using ImageJ software from NIH in the United States. The antibodies utilized in this research were: Dkk1 (21112-1-AP; 1:1000), WNT5A/B (55184-1-AP; 1:1000), Dvl3 (13444-1-AP; 1:1000), β-catenin (51067-2-AP; 1:1000), p-GSK-3β (67558-1-Ig; 1:1000), Collagen I (14695-1-AP; 1:1000), Collagen III (22734-1-AP; 1:1000), COL4 (17023-1-AP; 1:1000), fibronectin (15613-1-AP; 1:1000), α-SMA (14395-1-AP; 1:1000), ATG5 (10181-2-AP; 1:1000), ATG7 (67341-1-Ig; 1:1000), beclin1 (11306-1-AP; 1:1000), P62 (18420-1-AP; 1:1000), LC3 (14600-1-AP; 1:1000), ATF4 (10835-1-AP; 1:1000), and TFEB (13372-1-AP; 1:1000) purchased from Proteintech; p-RPS6KB (9234; 1:1000), RPS6KB (2708; 1:1000), EIF4E (2067; 1:1000), p-EIF4E (9741; 1:1000), MAPK (4695; 1:1000), p-MAPK (4370; 1:1000), AKT (4691; 1:1000), p-AKT (4060; 1:1000), goat anti-rabbit IgG (7074; 1:2000), and goat anti-mouse IgG (7076; 1:2000) purchased from Cell Signaling Technology; anti-TSG101 antibody (ab30871; 1:2000) purchased from abcam; GAPDH (ab9485; 1:2000) obtained from abcam.

### Histology and immunohistochemistry

A 4% paraformaldehyde solution (Solarbio, G1101) was used to fix human and mouse endometrial tissues and then embed in paraffin. Staining was carried out according to the kit instructions (Solarbio, G1348) with hematoxylin and eosin, or Masson’s Trichrome. Following removal of paraffin, rehydration, and quenching with 3% hydrogen peroxide and antigen retrieval. The sections were incubated with primary antibody overnight at 4 °C and then with HRP-conjugated secondary antibodies (Typng, 17,901,353–1) at room temperature for 30 min. The antigen signals were visualized using DAB substrates (Typng, TPB13). Hematoxylin counterstained sections were quantified using Image-Pro Plus software (Media Cybernetics, USA). The following antibodies were used in IHC: Dkk1 (21112-1-AP; 1:1000), P62 (18420-1-AP; 1:1000), LC3 (14600-1-AP; 1:1000), and TFEB (13,372-1-AP; 1:200) purchased from Proteintech; LAMP1 (9091; 1:200) was acquired from Cell Signaling Technology.

### Mice

Animals are raised in the Animal Center at Chongqing Medical University in accordance with the institutional guidelines for laboratory animals. The following mouse strains were used: Pgr^iresCre/+^ mouse < B6.129 S(Cg)-Pgr^tm1.1(cre)Shah^/AndJ (Stock No: 017915> ) purchased from Cyagen Biosciences (Suzhou, China) and Dkk1^flox/flox^ (Dkk1^f/f^) mouse provided by Cyagen Biosciences (Suzhou, China) [13, 14]. Uterine-specific gene knockout mice were generated by crossing Pgr^iresCre/iresCre^ male mice with Dkk1^fl/fl^ female mice, and uterine-specific gene knockout female mice were mated with fertile males to check their fertility. Based on Jackson Laboratory protocols, the tail DNA of these mice was genotyped.

### Immunofluorescence

The steps of immunofluorescence were referred to our previous studies. Briefly, after waxing, hydration, peroxidase quenching with 3% H2O2 and antigen retrieval, a primary antibody was incubated at 4 °C overnight in the dark on tissue sections, followed by a secondary antibody at room temperature. 30 min. Nuclei were stained with DAPI (Abcam, 104,139).

### Endometrial stromal cells

Human immortalized endometrial stromal cell line (T-HESCs, CRL-4003) was purchased from the American Type Culture Collection (ATCC). Primary human endometrial stromal cells (PHESC) were purchased from Procell Life Science&Technology Co., Ltd. (Cat NO.: CP-H233). The cells were cultured in DMEM/F12 medium without phenol red (Sigma) supplemented with 10% charcoal/dextran-treated fetal bovine serum (FBS, HyClone), 1% ITS (Gibco), 1.5 g/L sodium bicarbonate, and 500 ng/mL puromycin. Cell purity was verified by short tandem repeats (STR) profiling using PowerPlex 16 HS System (Promega) monthly. LookOut Mycoplasma polymerase chain reaction (PCR) Detection Kit (MP0035, Sigma-Aldrich) and JumpStart Taq DNA Polymerase (D9307, Sigma-Aldrich) were also used once a month to ensure that the cells were not contaminated by mycoplasma. ***Plasmids and siRNA transfection***.

Under 5% carbon dioxide, cells were incubated at 37 °C with oligonucleotides synthesized by Genepharma (Shanghai, China). The following sequences were targeted for human DKK1, WNT5A and TCF4 small interfering RNA (siRNA): DKK-1-1:5′-AGCACCUUGGAUGGGUAUUCTT-3′; DKK-1–2: 5′-GCCGGAUACAGAAAGAUCATT-3′; TCF4: 5′-UUCUUUGUCUGUACCUCCATT-3′; Wnt5a: 5′-UAACCCUGUUCAGAUGUCATT-3′; β-catenin: 5′-CAUGUGUUGGUAAGCUCUATT-3′; and NC (negative control) siRNA: 5′-UUCUUCGAAGGUGUCACGUTT-3′. DKK1-lentiviral expression vector (named LPP-U0513-DKK1) was provided by GeneCopoeia. Western blot analysis was performed after 72 h .

### Exosome purification

FBS (10%) was centrifuged overnight at 100,000 g to remove the bovine exosomes. A medium containing 10% exosome-free FBS was used to culture the cells. Exosomes were isolated by serial centrifugation as follows: cells were pelleted at 320 g for 10 min, and the supernatant was centrifuged for 15 min at 2,000 g for removing dead cells. After collecting the supernatant, it was ultracentrifuged (Beckman Coulter Optima L-100 XP, Beckman Coulter) for 30 min at 4℃. Exosomes were then clarified after ultracentrifugation at 100,000 g for 70 min at 4℃. The purified exosome pellets were obtained by another ultracentrifugation at 100,000 g for 70 min after PBS washing.

### Nanoparticle tracking analysis

Exosomes were diluted to 2 × 10^8^ ~ 20 × 10^8^ particles per milliliter before analysis, and relative concentrations were calculated based on dilution factors. The number and size distribution of exosomes were determined by measuring the Brownian motion rate using a NanoSight LM10 system (NanoSight, Amesbury, UK). Data analysis was performed using NTA 2.1 software (Nanosight).

### Cell proliferation experiment

We performed a cell proliferation assay using the Cell-Light Apollo567 In Vitro Kit (RiboBio, Guangzhou, China). Details are described in a previous study [15].

### Statistical analysis

Data were analyzed using GraphPad Prism Software, version 8 (GraphPad Software, Inc., La Jolla, CA). Data were presented as means ± SD. Unpaired student t-tests were used for continuous variables between two different groups. Analysis of variance (ANOVA) followed by the appropriate post hoc testing was used for comparisons among multiple groups. *P* < 0.05 represented statistical significance.

## Results

### DKK1 decreased in endometria of IUA patients

**Fig. 1:**
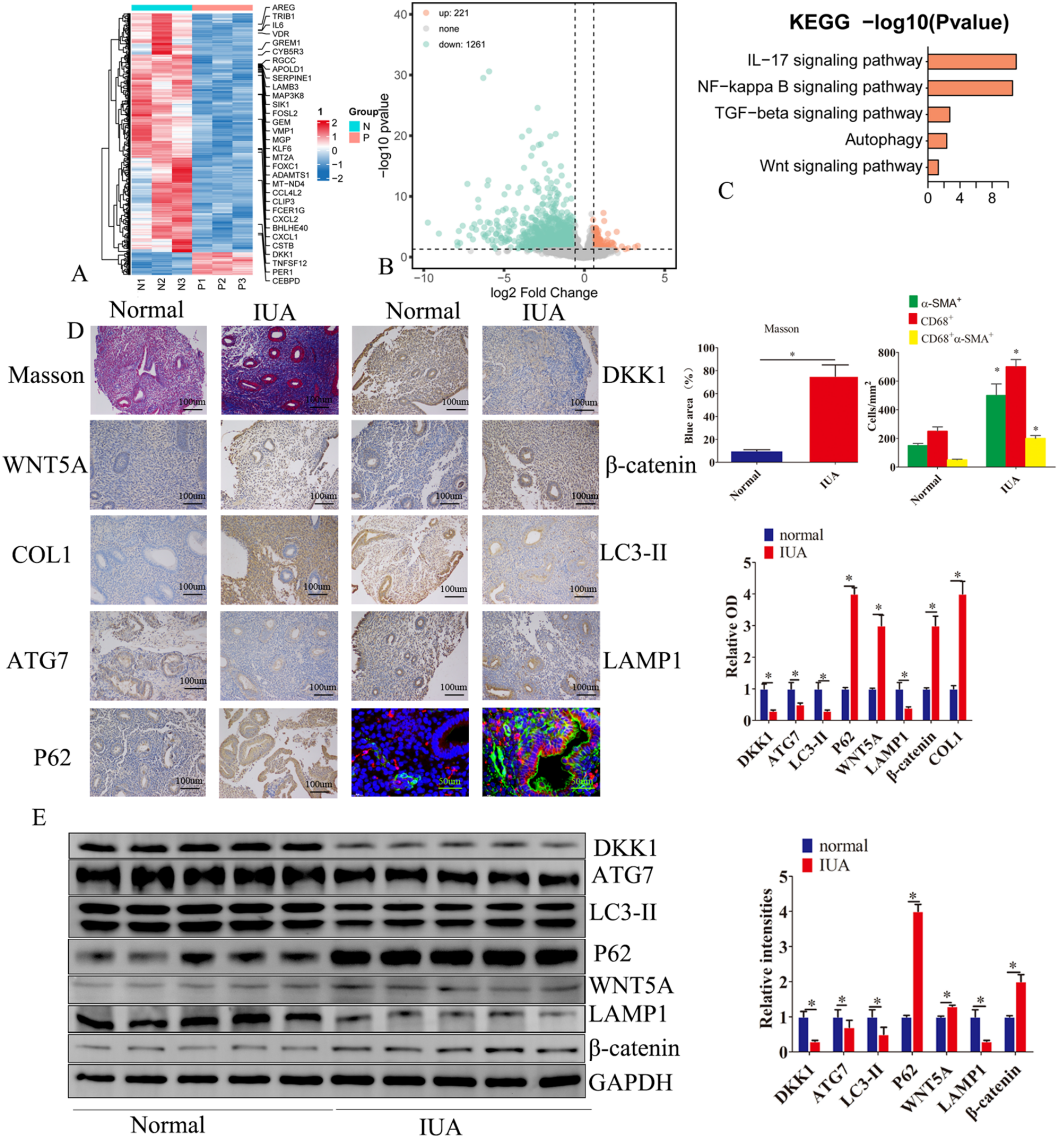
DKK1 was is decreased in endometria of IUA patients. (**A**) Next-generation sequencing was used to detect transcriptome differences between normal proliferating endometrial tissues and endometrium of patient with uterine adhesions. Figure A is a heat map. Figure (**B**) is a volcanic map. (**C**) KEGG signaling pathway analysis of differentially expressed genes. (**D**) Masson staining was used to detect the degree of endometrial fibrosis in normal endometrial and endometrium of patients with uterine adhesion. The expressions of DKK1, wnt5a, β-catenin, COL1, ATG7, LC3-II, LAMP1 and P62 in normal endometrium and endometrium of patients with uterine adhesion were detected by immunohistochemistry. Immunofluorescence detection of α-SMA (green) and CD68 (red) in normal endometrial and endometrial tissues of adhesion patients. (E)Western blot assay was used to detect the expression of DKK1, wnt5a, β-catenin, COL1, ATG7, LC3-II, LAMP1 and P62 in normal endometrium and endometrium in patients with uterine adhesions. Error bars represent the standard error. The symbols * and ** indicate *p* < 0.05 and 0.01, respectively. Scale bar: 5 μm

We used next-generation sequencing technology to detect normal endometrial tissues and the endometria of the transcriptome in IUA patients. A total of 1482 genes were differentially expressed between normal controls and IUA patients (fold change ≥ 2, *P* < 0.05)(Fig. 1A and B). KEGG analysis showed that the Wnt pathway, TGFβ pathway, IL-17 pathway, and autophagy pathway were abnormally expressed (Fig. 1C). Compared with normal endometrium, the expression of DKK1 mRNA was significantly down-regulated in the endometria of IUA patients (Fig. 1A). Immunohistochemical results indicated that DKK1 was expressed in both endometrial glandular epithelial and stromal cells (Fig. 1D). Also, compared with normal endometrium, the DKK1 protein level was decreased in the endometria of IUA patients (Fig. 1D and E). In addition, the expressions of ATG7, LAMP1, and LC3-II were decreased in endometria of IUA patients while the expressions of P62, wnt5A, COL1, and β-catenin were increased (Fig. 1D and E). We also observed increased expression of α-SMA and macrophages in the endometria of IUA patients (Fig. 1D). Masson staining revealed that endometrial fibrosis was enhanced in the endometria of IUA patients (Fig. 1D).

### Loss of DKK1 in the endometrium of mouse exhibited a fibrotic phenotype

**Fig. 2:**
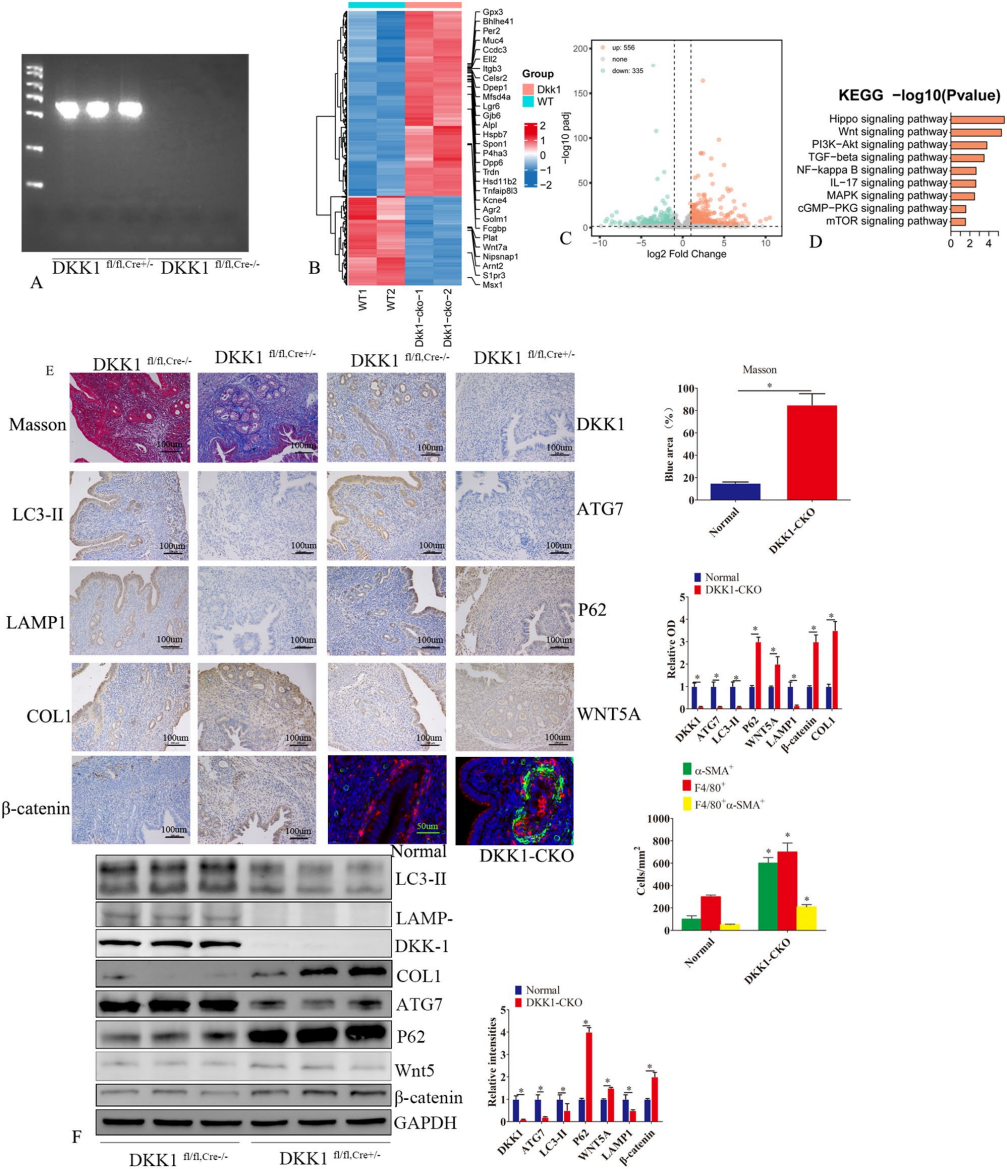
Loss of DKK1 in endometrium of mouse exhibited a fibrotic phenotype. (**A**) The homozygotes of DKK1 conditional knockout mice(DKK1^fl/fl, Cre+/−^) were identified by PCR. (**B**) Next-generation sequencing was used to detect transcriptome differences between endometrial tissues of DKK1^fl/fl, Cre+/−^ mice and endometrial tissues of DKK1^fl/fl, Cre−/−^ mice. Figure B is a heat map. Figure (**C**) is a volcanic map. (**D**) KEGG signaling pathway analysis of differentially expressed genes. (E) Masson staining was used to detect the degree of endometrial fibrosis between endometrial tissues of DKK1^fl/fl, Cre+/−^ mice and endometrial tissues of DKK1^fl/fl, Cre−/−^ mice. The expressions of DKK1, wnt5a, β-catenin, COL1, ATG7, LC3-II, LAMP1 and P62 were detected by immunohistochemistry between endometrial tissues of DKK1^fl/fl, Cre+/−^ mice and endometrial tissues of DKK1^fl/fl, Cre−/−^ mice. Immunofluorescence detection of α-SMA (green) and F4/80 (red) between endometrial tissues of DKK1^fl/fl, Cre+/−^ mice and endometrial tissues of DKK1^fl/fl, Cre−/−^ mice. Error bars represent the standard error. The symbols * and ** indicate *p* < 0.05 and 0.01, respectively. Scale bar: 5 μm

In order to investigate how DKK1 impacts endometrial fibrosis in vivo, we generated mice with conditional deletion of DKK1 in progesterone receptor (PGR)-positive cells. We named C57BL/6-Dkk1^flox/flox, PR−cre+/−^ mouse, with conditional knockout DKK1 in the endometrial tissues. The results of genotype detection showed that the *DKK1* gene in the endometrium was knocked out in homozygotes of DKK1 CKO mice (Fig. 2A). Western blot experiments further confirmed that the DKK1 protein levels were significantly down-regulated in DKK1 CKO homozygotes (Fig. 2E and F). Masson staining revealed that compared with C57BL/6-Dkk1^flox/flox, PR−cre−/−^ mice, the endometrial gland epithelium and stroma of C57BL/6-Dkk1^flox/flox, PR−cre+/−^ mice had obvious fibrosis type (Fig. 2E).

We used next-generation sequencing to analyze transcriptome expression in the endometrium of Dkk1 Dkk1^flox/flox, PR−cre+/−^ and Dkk1^flox/flox, PR−cre−/−^ mice. After knockout of DKK1 in mouse endometrium, we detected 891 differentially expressed genes (Fig. 2B). DKK1 CKO mice had an abnormal expression of the Wnt pathway, IL-17 pathway, PI3K-AKT pathway, and TGF-β pathway (Fig. 2D). Immunohistochemistry and Western blot showed that after DKK1 knockout, the expressions of ATG7, LAMP1, and LC3-II in mouse endometrial tissue were decreased and the expressions of P62, wnt5A, COL1, and β-catenin were increased (Fig. 2E and F). Immunofluorescence staining found that the expression of α-SMA and macrophages was increased in the endometrial tissue of DKK1 CKO mice (Fig. 2E), thus suggesting that macrophages may be involved in the occurrence of endometrial fibrosis.

### DKK1 promoted autophagy of endometrial stromal cells

**Fig. 3:**
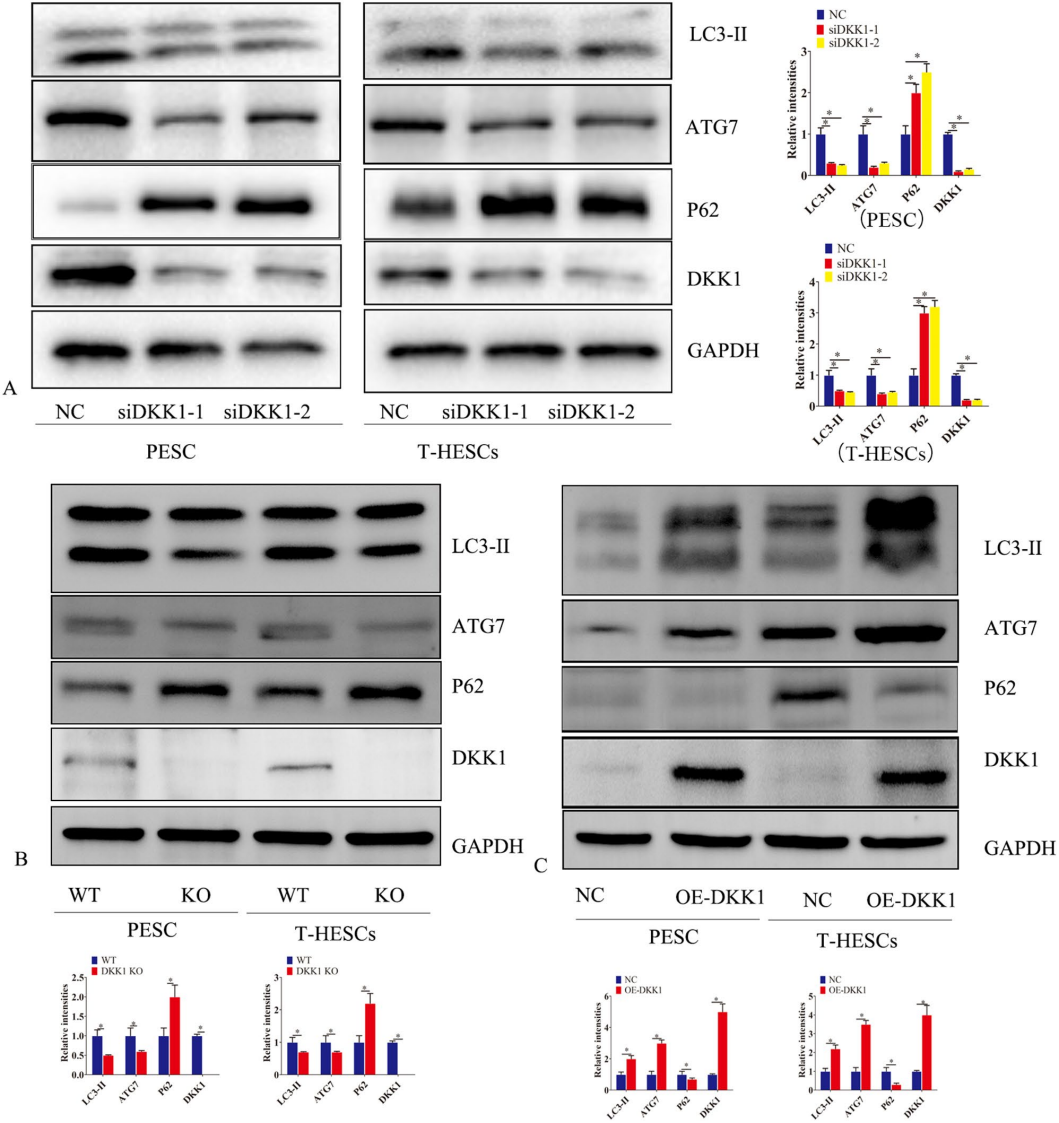
Dkk1 promoted autophagy of endometrial stromal cells. (**A**) After silencing DKK1 in primary endometrial stromal cells (PESC) and endometrial stromal cells immortalized cell lines (T-HESCS), western blot analysis was performed to detect the expression of DKK1,LC3-II, ATG7 and P62 proteins. (**B**)After knock out of DKK1 in primary endometrial stromal cells (PESC) and endometrial stromal cells immortalized cell lines (T-HESCS), western blot analysis was performed to detect the expression of DKK1,LC3-II, ATG7 and P62 proteins. (**C**) After ectopic expression of DKK1 in primary endometrial stromal cells (PESC) and endometrial stromal cells immortalized cell lines (T-HESCS), western blot analysis was performed to detect the expression of DKK1,LC3-II, ATG7 and P62 proteins. Error bars represent the standard error. The symbols * and ** indicate *p* < 0.05 and 0.01, respectively

It has been reported that DKK1, as an inhibitor of the canonical wnt pathway, is involved in regulating autophagy [16]. Our results showed that in human immortalized endometrial stromal cell lines (T-HESCs) and primary endometrial stromal cells, the expression of DKK1 was reduced after DKK1 silencing (Fig. 3A). After overexpression of DKK1, DKK1 protein and mRNA levels increased (Fig. 3C). After silencing DKK1, LC3-I to LC3-II conversion, ATG7 expression was reduced while P62 expression was increased (Fig. 3A). In T-HESCs and primary endometrial stromal cells, after DKK1 knockout, DKK1 protein levels were decreased. LC3-I to LC3-II conversion, ATG7 expression was reduced; P62 expression was increased (Fig. 3B). After overexpression of DKK1, the opposite phenomenon was observed (Fig. 3C).

### DKK1 regulates Wnt/β-catenin and PI3K/AKT/mTOR pathways

**Fig. 4:**
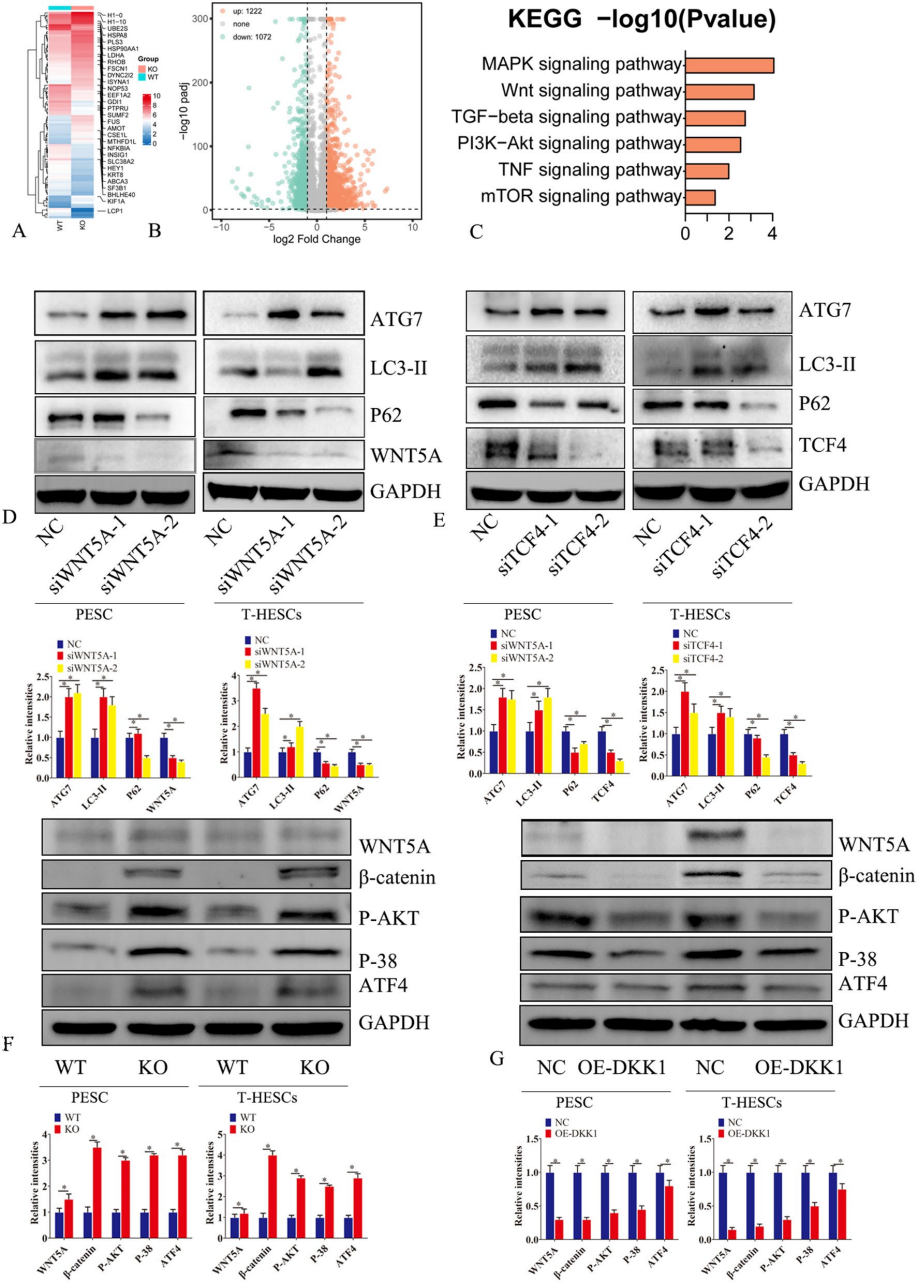
DKK1 regulates Wnt/β-catenin and PI3K/AKT/mTOR pathways. (**A**) After knock out of DKK1 in primary endometrial stromal cells, next-generation sequencing was used to detect transcriptome differences between KO group and WT group. Figure (**A**) is a heat map. Figure (**B**) is a volcanic map. (**C**) KEGG signaling pathway analysis of differentially expressed genes. (**D**) After silencing Wnt5a in primary endometrial stromal cells (PESC) and endometrial stromal cells immortalized cell lines (T-HESCS), western blot analysis was performed to detect the expression of Wnt5a, LC3-II, ATG7 and P62 proteins. (**E**) After silencing TCF4 in primary endometrial stromal cells (PESC) and endometrial stromal cells immortalized cell lines (T-HESCS), western blot analysis was performed to detect the expression of TCF4, LC3-II, ATG7 and P62 proteins. (**F**) After knock out of DKK1 in primary endometrial stromal cells (PESC) and endometrial stromal cells immortalized cell lines (T-HESCS), western blot analysis was performed to detect the expression of WNT5A, β-catenin, p-akt, p38 and ATF4 proteins. (**G**) After ectopic expression of DKK1 in primary endometrial stromal cells (PESC) and endometrial stromal cells immortalized cell lines (T-HESCS), western blot analysis was performed to detect the expression of WNT5A, β-catenin, p-akt, p38 and ATF4 proteins. Error bars represent the standard error. The symbols * and ** indicate *p* < 0.05 and 0.01, respectively

To investigate the effects of DKK1 on the downstream signaling pathways, we analyzed the transcriptome expression of DKK1 KO in endometrial immortalized cell lines, finding that the Wnt/β-catenin pathway, TGFβ pathway, as well as PI3K/AKT pathway were aberrated after DKK1 KO (Fig. 4A-C). Wnt/β-catenin has been reported to regulate autophagy. The knockdown of WNT5A and TCF4 reduced the expression of ATG7, LC3-II, WNT5A, and TCF4 in primary endometrial stromal cells and immortalized cells (Fig. 4C-D). DKK1 KO increased the expression of WNT5A and CTNNB1 in primary endometrial stromal cells and immortalized cells, while DKK1 overexpression decreased the expression of WNT5A and CTNNB1 (Fig. 4E-F). Dkk1 KO increased the expression of ATF4, p-AKT, and p-38, the substrates of MTOR, while overexpression of DKK1 resulted in opposite results (Fig. 4E-F), thus indicating that DKK1 regulates autophagy through Wnt and mTOR pathways.

### DKK1 KO promotes the release of exosomes

**Fig. 5:**
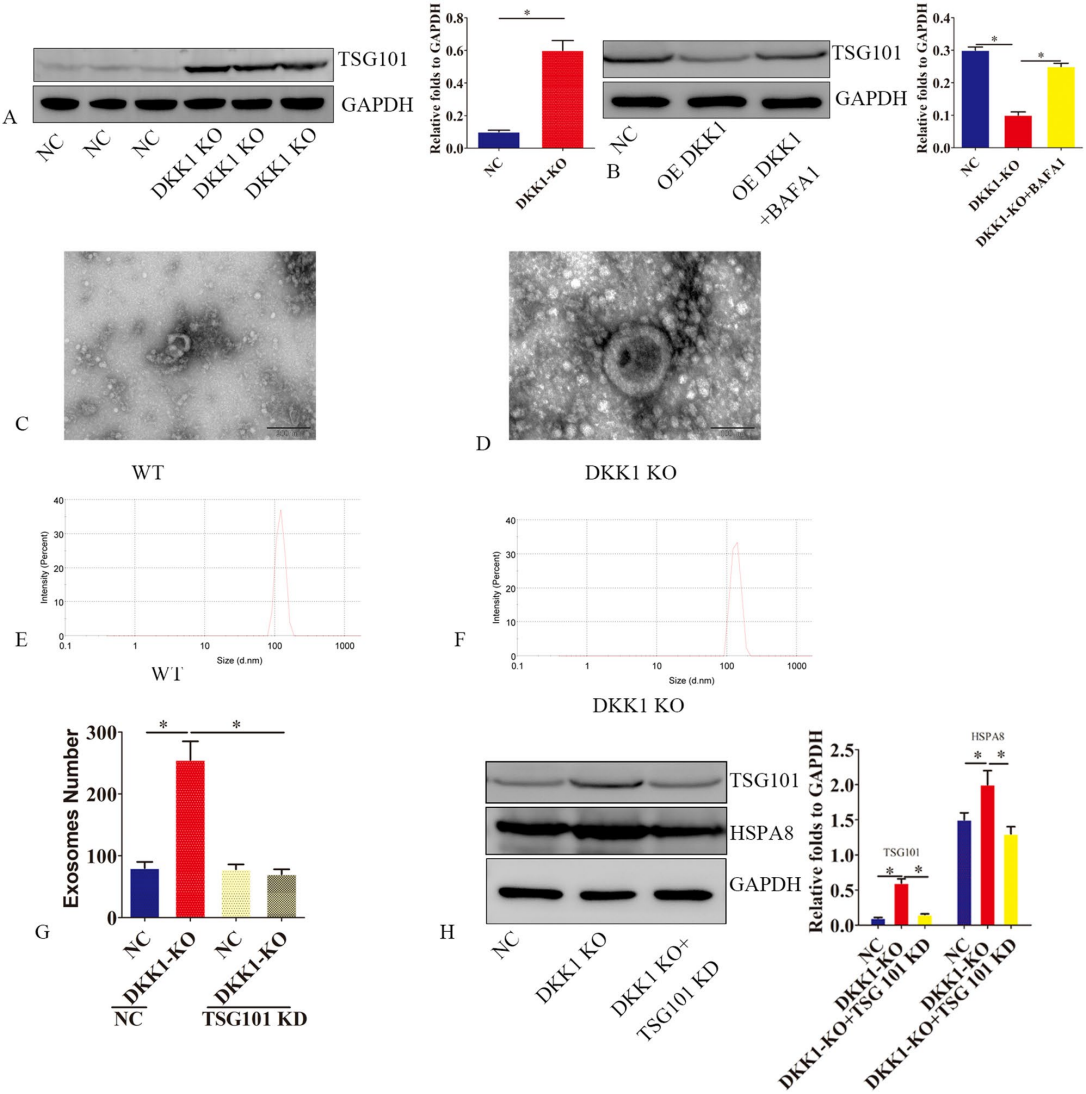
DKK1 ko promotes the release of exosomes. (**A**) The expression of TSG101 protein in primary endometrial stromal cells (PESC) was detected by western blot after DKK1 gene was knocked out. (**B**) primary endometrial stromal cells (PESC) with DKK1 overexpression were treated with bafa1. After 24 h, TSG101 protein was detected by western blot. Wild-type primary endometrial stromal cells (PESC) (**C**, **E**) and PESC with dkk1 gene knockout (**D** and **F**)were used to detect exosome morphology and diameter. (**G**) Quantitative analysis of primary endometrial stromal cells (PESC)-derived exosomes by ELISA. Data are the means ± SEM of 3 assays. (**H**) The expression of exosomal markers TSG101 and HSPA8 was detected by western blotting. Error bars represent the standard error. The symbols * and ** indicate *p* < 0.05 and 0.01, respectively

Considering that autophagy disorders can cause exosome release, we speculated whether the block of autophagic flow caused by DKK1 KO increased the secretion of exosomes from endometrial stromal cells. As ESCRT is MVB formation and secretion, we examined the effect of DKK1 KO on TSG101, finding that TSG101 expression was increased after KO (Fig. 5A). Overexpression of DKK1 promoted TSG101 degradation in CHX-treated primary endometrial stromal cells. However, it was delayed by bafa1 (Fig. 5B), which suggests that DKK1-mediated autophagy promotes TSG101 degradation.

To clarify the involvement of DKK1 in releasing exosomes, we extracted them using an exosome kit, and the presence of exosomes was clarified by electron microscopy (Fig. 5C-F). ELISA was used to detect CD63, revealing that the number of exosomes was increased by 2.5-fold in DKK1 KO cells; however, this increase was suppressed by silencing TSG101 (Fig. 5G). To determine whether TSG101 deficiency affected exosomal secretion, we found that exosomal markers TSG101 and HSPA8 were significantly increased in purified exosomes from DKK1 ko endometrial stromal cells, but this increase was reversed entirely by TSG101 silencing (Fig. 5H), thus suggesting that DKK1-mediated exosome secretion is TSG101 dependent.

### DKK1 exosomes promoted the transformation of macrophages into myofibroblasts

**Fig. 6:**
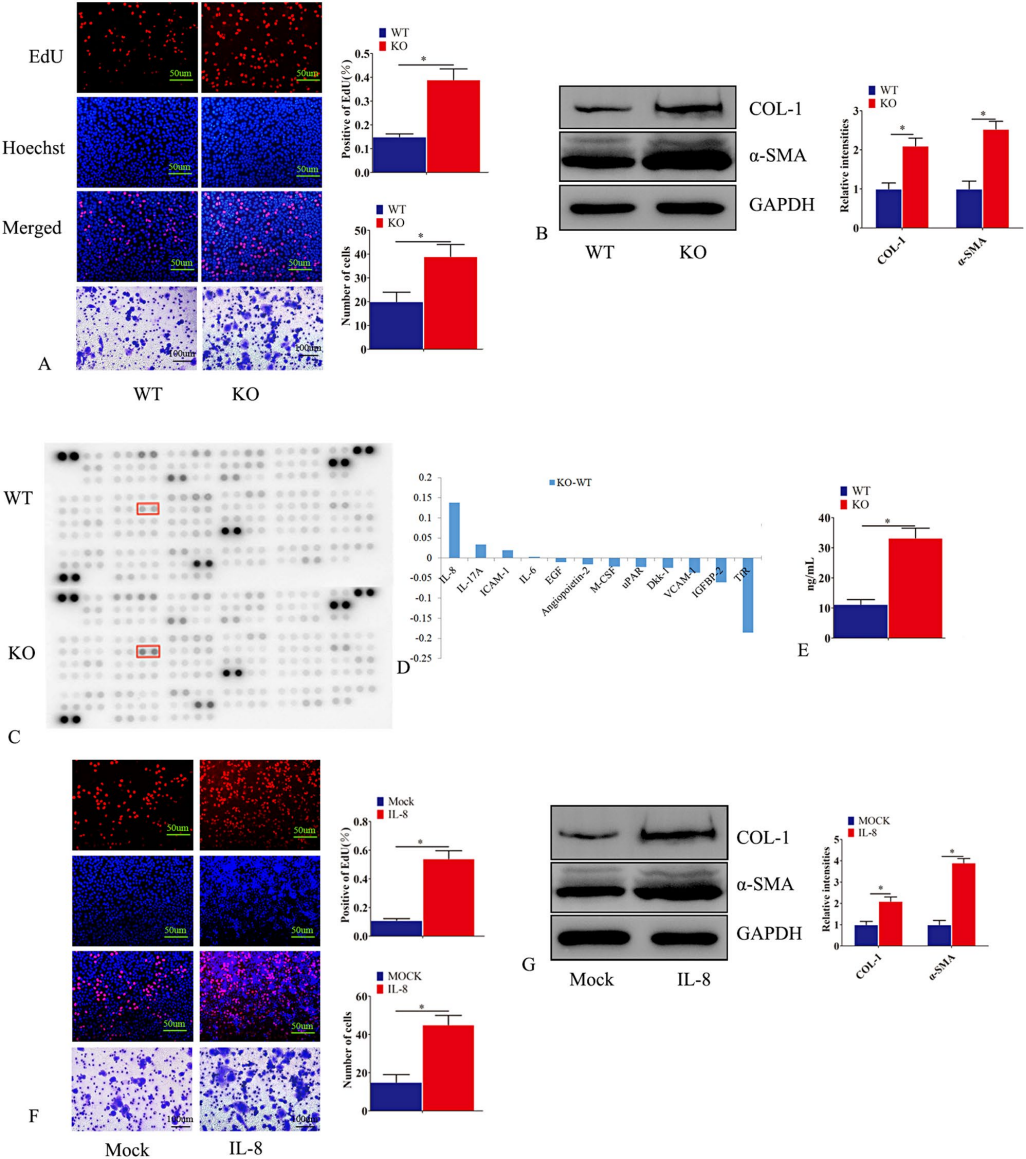
DKK1 exosomes promoted the transformation of macrophages into myofibroblasts. (**A**-**B**) Exosomes were extracted from Wild-type primary endometrial stromal cells (PESC) and PESC with DKK1 gene knockout. 72 h after exosomes were added to human macrophages, EdU assay was used to detect macrophage proliferation, and transwell assay was used to detect macrophage invasion status. The protein expressions of col1 and α-SMA were detected by Western blot. (**C**-**D**) Exosomes were extracted from Wild-type primary endometrial stromal cells (PESC) and PESC with DKK1 gene knockout. Cytokine antibody microarray was used to detect the expression of cytokines in exosomes derived from wild-type and DKK1 KO endometrial stromal cells. Knockout of DKK1-derived exosomes in endometrial stromal cells significantly increased the expression of IL-8 and IL-17 A. (**E**) elisa was used to detect the expression of IL-8 in exosomes derived from wild-type endometrial primary stromal cells or endometrial primary stromal cells with DKK1 gene knockout. (**F**-**G**) IL-8 was added to human macrophages for 72 h, EdU assay was used to detect macrophage proliferation, and transwell assay was used to detect macrophage invasion status. The protein expressions of col1 and α-SMA were detected by Western blot. Error bars represent the standard error. The symbols * and ** indicate *p* < 0.05 and 0.01, respectively. Scale bar: 5 μm

Macrophage-to-myofibroblast transition (MMT) is involved in the occurrence of a variety of fibrotic diseases. The MMT was also observed in human IUA specimens. Therefore, we observed the effect of exosomes produced by endometrial stromal cells on macrophages after DKK1 KO, finding that treatment of macrophages with exosomes derived from primary endometrial stromal cells DKK1 KO for 72 h enhanced macrophage proliferation and metastasis (Fig. 6A). However, this change was not observed in the control group. Furthermore, Western blotting showed that the expression of α-SMA and COL1 was increased after DKK1 KO endometrial stromal cells exosomes treated macrophages compared with the control group (Fig. 6B). Cytokine microarray was used to detect the expression of cytokines in exosomes. We found that IL-8 and IL-17 A were significantly increased after DKK1 KO in primary cells compared with control cells (Fig. 6C-E). After treatment of macrophages with IL-8, we also found that IL-8 could promote the proliferation and migration of macrophages (Fig. 6F). Western blot showed that the expression of α-SMA and COL1 increased after IL-8 treatment of macrophages (Fig. 6G).

### Autophagy activator inhibited endometrial fibrosis in DKK1 CKO mice

**Fig. 7:**
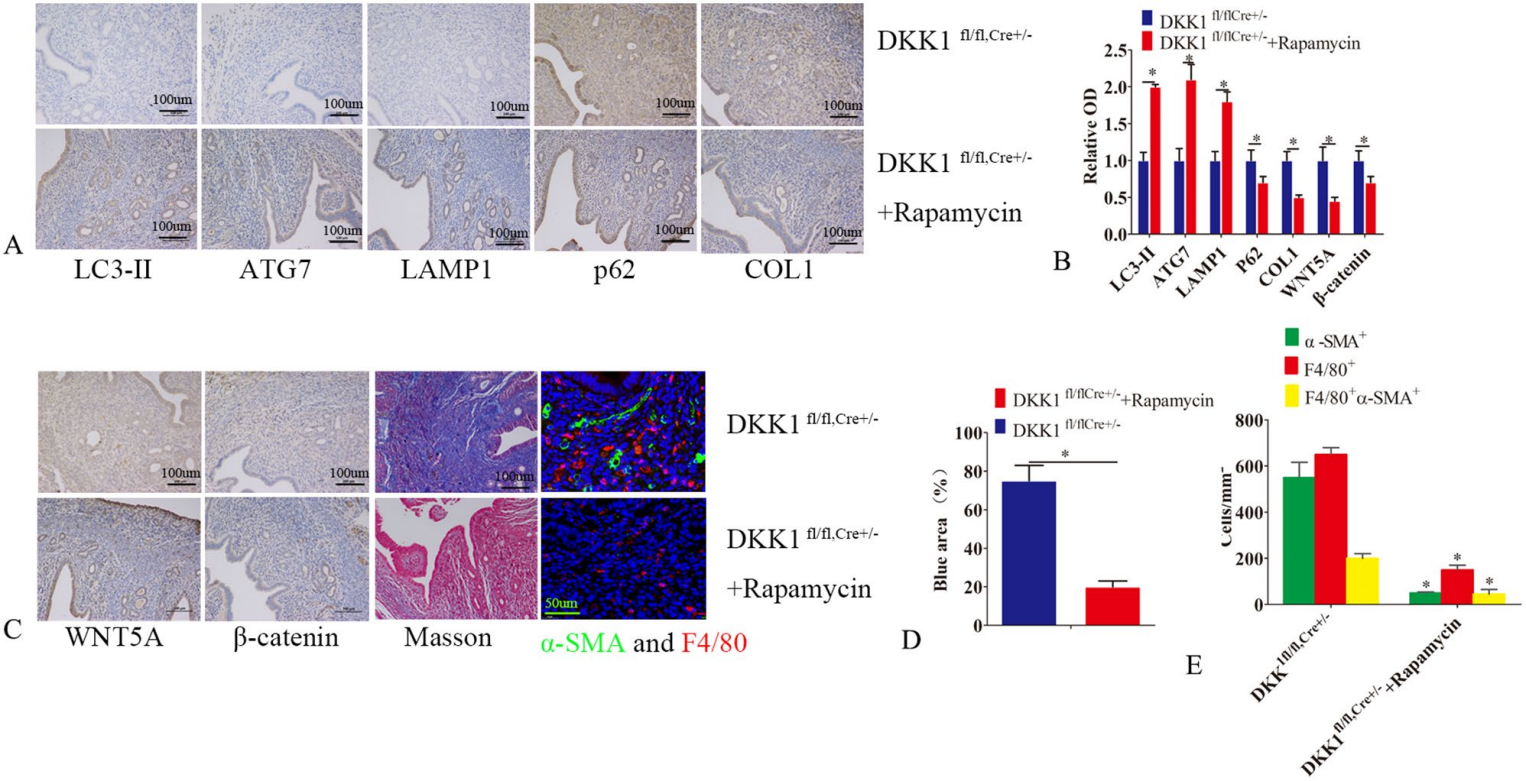
Autophagy activator inhibited endometrial fibrosis in DKK1 cko mice. (**A**) DKK1^fl/fl, Cre+/−^ mice were randomly divided into two groups. Rapamycin treatment group was given intraperitoneal injection of rapamycin(3 mg/kg/day). The control group was injected with equal volume of DMSO. Two weeks after administration of rapamycin, the mice were sacrificed. (**A**-**E**) Masson staining was used to detect the degree of endometrial fibrosis in rapamycin treatment group and control group. The expressions of DKK1, wnt5a, β-catenin, COL1, ATG7, LC3-II, LAMP1 and P62 in rapamycin treatment group and control group were detected by immunohistochemistry. Immunofluorescence detection of α-SMA (green) and F4/80 (red) in rapamycin treatment group and control group. Error bars represent the standard error. The symbols * and ** indicate *p* < 0.05 and 0.01, respectively. Scale bar: 5 μm

Two weeks after administration of rapamycin, DKK1 CKO homozygotes had increased endometrial glands and glandular epithelial cells. Masson staining revealed reduced fibrosis in CKO homozygotes (Fig. 7C). Immunohistochemistry showed that the expressions of LC3-II, ATG7, LAMP1, and P62 were increased in CKO homozygotes (Fig. 7A-C) while the expressions of WNT5A, β-catenin, and COI were decreased (Fig. 7A-C). In addition, immunofluorescence showed that macrophages and α-SMA localization were decreased (Fig. 7C and E), which suggests that autophagy activator can be used as a therapeutic target.

## Discussion

In the present study, we found that DKK1 and autophagy markers were down-regulated, and α-SMA and macrophage localization were increased in the endometrium of patients with IUA. DKK1 knockout mice showed a fibrotic phenotype with decreased autophagy and increased localization of α-SMA and macrophages in the endometrium. In vitro studies revealed that DKK1 knockout inhibited the autophagic flow of endometrial stromal cells. However, overexpression of DKK1 showed the opposite phenotype. DKK1 regulates autophagic flow through Wnt/β-catenin and PI3K/AKT/mTOR pathways. Further studies showed that DKK1 gene knockout promoted the secretion of IL-8 in exosomes, thereby promoting macrophage proliferation and metastasis. In DKK1 KO mice, treatment with autophagy activator rapamycin inhibitor partially restored the endometrial fibrosis phenotype. Our findings suggest that DKK1 may represent a potential diagnostic marker or therapeutic target for IUA.

As one of the inhibitors of the Wnt signaling pathway, DKK-1 protein can lead to rapid endocytosis by competitively binding to lipoprotein receptor-associated protein 5/6 (LRP 5/6) or by forming a triplet complex with transmembrane proteins Kremen and LRP 5/6, reduction of plasma membrane LRP 5/6 and inhibition of Wnt signaling pathway [17–19]. Wnt signaling and Wnt/beta-catenin target genes are activated in a mouse model of renal interstitial fibrosis [20, 21]. Administration of Wnt antagonist Dickkopf-1 significantly reduces renal beta-catenin accumulation and inhibits the expression of Wnt/beta-catenin target genes. At the same time, Dickkopf-1 can inhibit myofibroblast activation [22, 23]. In addition to inhibiting the Wnt pathway by competitive binding to coreceptor LRP 5/6, DKK-1 can also prevent its transformation into myofibroblasts by inhibiting the MAPK and JNK signaling pathways mediated by PDGF, TGF-β, and connective tissue growth factor, thereby delaying the process of fibrosis [24]. Our results showed that DKK1 expression was significantly reduced, and fibrosis was enhanced in the endometrium of human patients with IUA. In DKK1 KO mouse endometrium, the endometrium showed fibrosis phenotype, thus suggesting that loss of DKK1 leads to endometrial fibrosis. Therefore, the decrease of DKK1 may cause intrauterine adhesion, and DKK1 may be a potential target for treating IUA.

As an inhibitory molecule of the Wnt/β-catenin signaling pathway, DKK1 promotes autophagy. DKK1 can promote autophagy through the PI3K/AKT/mTOR signaling pathway. Our study found that DKK1 could inhibit Wnt//β-catenin and PI3K/AKT/mTOR signaling pathways, thereby inducing autophagy, which is consistent with previous reports [16]. Previous studies have linked autophagy to exosome secretion [25, 26]. Researchers have shown in fibroblasts that autophagosomes can fuse with endosomes to form twosomes. Twosomes are then directly fused with lysosomes and degrade endosomal contents [27]. A dysfunctional autophagy pathway leads to the specific endosome of MVBs not being oriented toward the autophagy pathway, but instead joining with plasma membranes and releasing contents into exosomes to maintain intracellular homeostasis [28]. In the present study, DKK1 KO was found to increase the accumulation of SQSTM1, which was accompanied by the increase of exosomes and the inhibition of autophagic flux in endometrial stromal cells. In endometriosis, inhibition of IL-8 suppresses endometrial fibrosis and inflammation [29]. Interleukin-8 has been found to promote carbon tetrachloride (CCl4)-induced liver fibrosis through the PI3K/Akt/HIF-1α pathway [30]. IL-8 promotes fibrosis in idiopathic pulmonary fibrosis mesenchymal progenitor cells. The mechanism is based on interleukin-8 recruiting activated macrophages into fibrotic lesions [31]. This study found that DKK1 reduction led to increased interleukin-8 in exosomes. Interleukin-8 can promote macrophages’ proliferation, migration, and transformation into myofibroblasts. Thus, loss of DKK1 leads to an increase in exosomes and an increase in interleukin-8, which causes the transformation of macrophages into myofibroblasts and finally leads to fibrosis of the endometrium.

The role of autophagy in fibrotic diseases is controversial, and it can promote or inhibit the occurrence of fibrotic diseases. In IUA, autophagy markers were reduced. Activation of autophagy can inhibit endometrial fibrosis. Inhibition of autophagy can lead to the occurrence of endometrial fibrosis. At the same time, activation of autophagy can inhibit the occurrence of EMT in endometrial epithelial cells [11]. Our study found that the expression of autophagy markers was decreased in the endometrium of patients with IUA, which is consistent with previous reports. In addition, autophagy inducer rapamycin partially inhibited endometrial fibrosis in DKK1 KO mice, thus suggesting that activation of autophagy can be used as a therapeutic target for IUA.

Macrophage-myofibroblast transition (MMT) can promote the metastasis of malignant tumors and the occurrence of fibrotic diseases [32]. MMT promotes interstitial fibrosis in chronic renal allograft injury and SMAD3 signaling is involved in this process [33]. As a target of SMAD3, Pou4f1 leads to renal fibrosis by promoting MMT [33]. The proto-oncogene tyrosine-protein kinase Src is essential for the macrophage to myofibroblast transition during renal scar formation [34]. In the present study, we found that α-SMA and macrophage localization were increased in the endometrium of patients with IUA, which indicated that MMT is involved in the occurrence of intrauterine adhesion. α-sma and macrophage localization were also increased in DKK1 KO mice, thus suggesting that DKK1 KO can promote MMT. Our study revealed that decreased DKK1 expression in IUA may contribute to endometrial fibrosis through MMT.

**Fig. 8:**
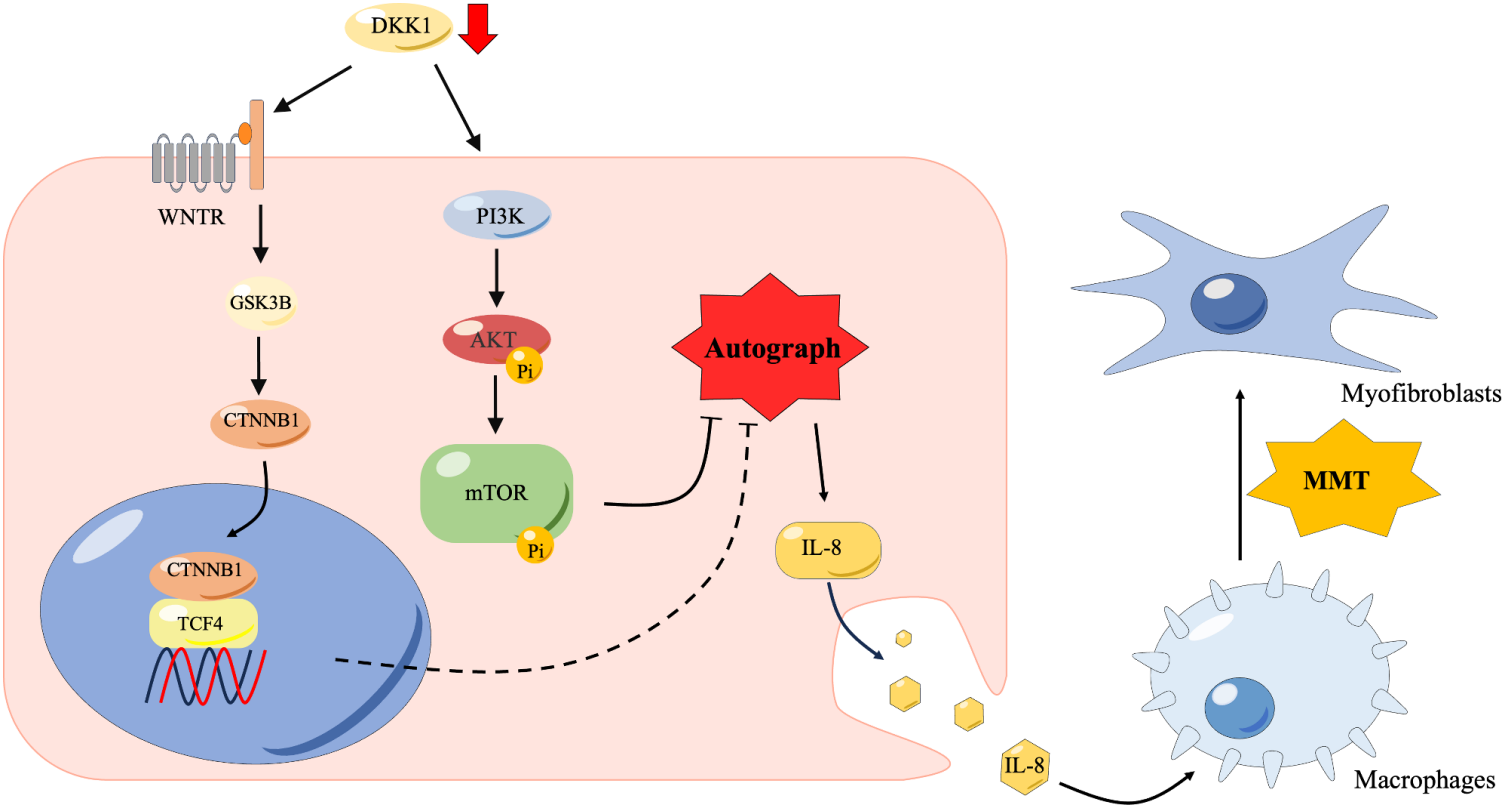
The graphic abstract of this study

In conclusion, we have shown for the first time that DKK1 expression is decreased in the endometrium of patients with IUA, and its reduction can lead to the development of endometrial fibrosis. At the same time, the decrease of DKK1 can lead to the block of autophagy, the increase of exosomes, the increase of IL-8 secretion, and the transformation of macrophages into myofibroblasts (Fig. 8). DKK1 can be used as a diagnostic molecular marker and a potential therapeutic target for IUA. In this research, findings from in vitro and animal models do not always translate effectively to human clinical settings, potentially limiting the applicability of the results for therapeutic interventions. Therefore, we will detect the expression of DKK1 in patients’ blood samples and explore the clinical value of DKK1 in the diagnosis of uterine adhesions. At the same time, we will also try to study the potential value of DKK1 synthetic protein in the treatment of uterine adhesion.

### Electronic supplementary material

Below is the link to the electronic supplementary material.

Supplementary Material 1

Supplementary Material 2
