# Supplementary figures and images for: DKK1 loss promotes endometrial fibrosis via autophagy and exosome-mediated macrophage-to-myofibroblast transition
Source: J Transl Med. 2024 Jul 3;22:617. doi: 10.1186/s12967-024-05402-5 (PMC11223343; doi:10.1186/s12967-024-05402-5)

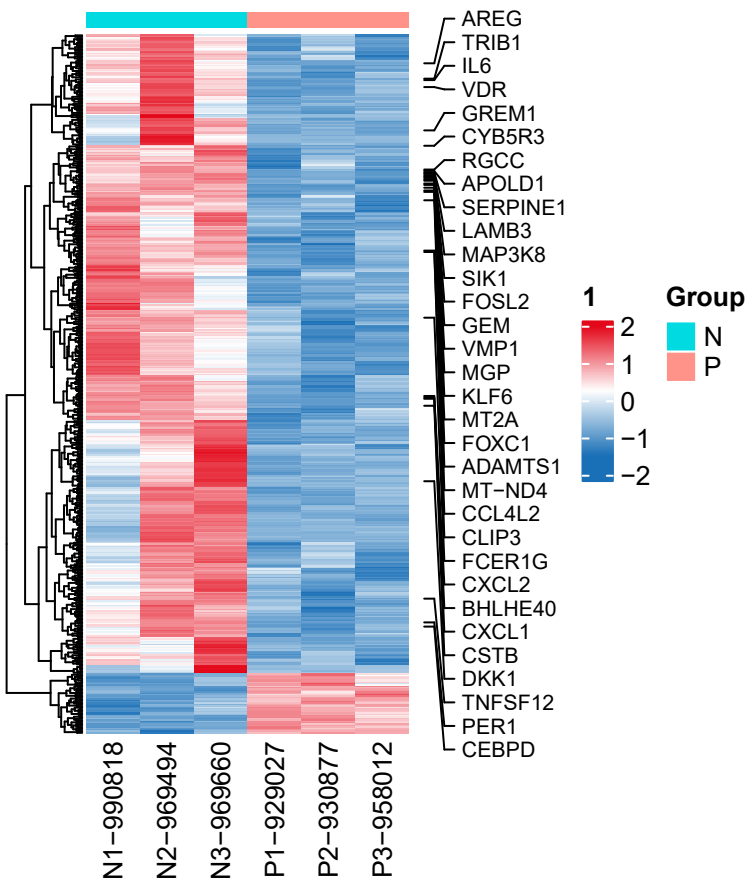

Supplement: Supplementary file 1 — Supplementary Material 1 [file 12967_2024_5402_MOESM1_ESM.pdf]
